# Epidemiological Situation of High-Prevalence Non-Communicable Diseases in Spain: A Systematic Review

**DOI:** 10.3390/jcm12227109

**Published:** 2023-11-15

**Authors:** Yessineth D. Aparicio-Rodríguez, Enrique Alonso-Morillejo, Juan Manuel García-Torrecillas

**Affiliations:** 1 Faculty of Health Sciences, University of Almería, 04120 Almería, Spain; ealonso@ual.es; 2 Emergency and Research Unit, Torrecardenas University Hospital, 04009 Almería, Spain; garcia.torrec@yahoo.es; 3 CIBER de Epidemiología y Salud Pública (CIBERESP), 28029 Madrid, Spain; 4 Instituto de Investigación Biosanitaria Ibs, 18012 Granada, Spain

**Keywords:** chronic non-communicable diseases, colorectal cancer, ischaemic heart disease, type 2 diabetes mellitus, risk factors, healthy living, incidence, prevalence, mortality

## Abstract

High-prevalence non-communicable diseases (HNCDs) are an ongoing global public health problem, posing a risk to the continuity of the 2030 Agenda for Sustainable Development. The aim of this study is to describe the current situation in Spain regarding certain HNCDs, namely, ischaemic heart disease, type 2 diabetes mellitus and colorectal cancer, including their prevalence and incidence in recent years. A systematic review was conducted between October 2022 and February 2023 using the MEDLINE, ProQuest and Scopus databases. After an exhaustive search, a total of thirty-four articles were included, comprising fourteen articles on colorectal cancer, seven on ischaemic heart disease and thirteen on diabetes mellitus type 2. The main topics included risk factors, lifestyles, mortality and incidence, the importance of screening and patient empowerment. On analysing each disease, it can be gleaned that risk factors and lifestyle impact the incidence, prevalence and mortality of the diseases studied. In addition, responsible human behaviour, associated with lifestyle factors, is related to the occurrence of these three diseases.

## 1. Introduction

Globally, chronic non-communicable diseases (HNCDs) account for 74% of deaths, or 41 million, each year [1]. According to the World Bank (2020) [2], the rise of HNCDs has become a global emergency. Consequently, they represent a threat to the continuity of the 2030 Agenda for Sustainable Development goal, whose primary aim is to reduce the likelihood of death from major HNCDs by one-third [1].

According to the World Health Organization (WHO) (2022) [1], cardiovascular diseases are responsible for the highest number of yearly NCD mortalities, accounting for 17.9 million people, followed by cancers at 9.3 million, chronic respiratory diseases at 4.1 million and diabetes at 2.0 million. In addition, HNCDs may lead to an increase in demand for health services, both because of the cost of treatment and the increase in public health expenditure they bring about [3]. 

A study by the United Nations (UN) (2020) [4] claimed that in 2020, approximately one billion people spent 10% of their household income on health care, with lower-middle-income countries being the most affected, according to the World Bank classification [5]. In the European Union, HNCDs account for 25.7% of healthcare budgets [6]. Consequently, NCD costs are high and are expected to rise sharply in the European Union [7]. However, it should be remembered that there are grants, such as those from the World Bank, that provide financial support, advice and medical assistance through governments, allowing them to greatly strengthen their health systems and address risk factors more efficiently [2].

As previously stated, it is estimated that by 2030, NCD mortality will be reduced by one-third through prevention and health promotion tools [8]. Studies such as Ángel et al. (2018) [9] recognise the central importance of educational work, as well as promotion and prevention activities, in tackling HNCDs. Other factors such as communication, organisation, leadership, evaluation, planning and financial stability are also vital when implementing an NCD programme [10]. The World Health Organization (2022) [11] states that NCD indicators are intended to encourage countries to align their policies to prevent and control HNCDs. Therefore, policy guidelines should be equity-oriented, with the strategies implemented regarding the risk factors for the main HNCDs having a scientific and analytical basis [12]. 

In Spain, the National Health System (SNS) is responsible for providing free and universal health care to the Spanish population, taking a comprehensive approach to ensure health is maintained [13]. However, the Ministry of Health (2021) [14] states that 34% of the population experiences at least one chronic problem during their lifetime, with people over 65 (77.6%) being the most affected. According to data from the World Health Organization’s NCD report (2022) [15], the population of Spain has a total population of 46,737,000 people, with 387,300 deaths caused by HNCDs and a 10% risk of premature death from HNCDs. Data collected by the National Institute of Statistics (INE) (2022) [16] up to 2021 reported that 26.4% of deaths were due to diseases of the circulatory system, 25.2% were due to tumours and 10.2% were due to infectious diseases. 

In this study, we analysed three diseases that are at the core of HNCDs: ischaemic heart disease, diabetes mellitus and colorectal cancer (CRC). All of them account for a considerable percentage of the mortality rate in Spain, are highly prevalent and are a leading cause of mortality in the country. Ischaemic heart disease (IHD) had an incidence in 2019 of 361.4 new cases per 100,000 population and was substantially higher in men (463.4) than in women (263.6). Its prevalence has risen over the last 10 years, from 2.8% in 2009 to 3.3% in 2019 [17]. Secondly, we addressed diabetes mellitus, specifically type 2 diabetes. In its tenth edition, the International Diabetes Federation (IDF) (2021) [18] announced that in 2021, around 537 million people were suffering from diabetes in the world. According to the report by the Ministry of Health (2017) [19], there were 3.07 million people with diabetes in Spain, with a prevalence of 6.8% and a frequency of 3.4% for type 1 diabetes and 96.6% for type 2. However, this has been increasing as the current prevalence is 14.8%, which is the second highest in Europe [20]. We also studied the situation regarding colorectal cancer (CRC) with the International Agency for Research on Cancer (IARC) (2021) [21], establishing, through the main source of cancer data, Cancer Today, that Spain had a prevalence of 858,220 cases in the period 2015–2020 [22]. These new cases were reflected as follows: 14.3% for colorectal cancer, 12.3% for prostate cancer, 12.1% for breast cancer, 10.3% for lung cancer, 6.6% for bladder cancer and 44.5% for other cancers. Consequently, an increase in cancer incidence and mortality is predicted worldwide in the coming years, with Spain in particular going from 112,000 cases in 2020 to 159,000 cases in 2040 [23].

As time goes by, it is increasingly evident that these chronic diseases continue to be a global and regional public health problem. However, it is remarkable how their prevalence and incidence are increasing despite the existence of programmes and care plans designed to combat the risk factors inherent to each disease. This is why a current evaluation of these diseases will allow us to explore the situation in Spain and thus provide new findings that will lead to the search for improvements in public health regarding these HNCDs. Thus, through a systematic review, our objective is to describe the current situation in Spain regarding certain HNCDs, namely, ischaemic heart disease, diabetes mellitus type 2 and colorectal cancer, including their prevalence and incidence in recent years.

## 2. Materials and Methods

### 2.1. Protocol and Registration

The methodological approach carried out was a systematic review of the literature between October 2022 and February 2023 on the current situation in Spain concerning three HNCDs, namely, ischaemic heart disease, diabetes mellitus type 2 and colorectal cancer. This review followed the guidelines of PRISMA (Preferred Reporting Items for Systematic reviews and Meta-Analyses) Page et al. (2021) [24] and also made use of the International Prospective Register of Systematic Reviews (PROSPERO), no. CRD42023425622. Our research question was structured using the tools provided by the PICO mnemonic (see Appendix A).

### 2.2. Inclusion and Exclusion Criteria

The inclusion criteria took into account articles published in the period 2022–2023 that contained epidemiological information relating to Spain in the last 5 years in Spanish and English. In addition, we included studies from Spain that referred to variables such as age (over 18 years of age), sex, autonomous communities, disease risk factors, economic status, education, health status, hospitalisation and mortality rate. 

As such, we excluded studies that had been published more than five years ago, that did not belong to Spain, those with a study sample of people under 18 years of age and those that did not refer to the variables investigated in this study. 

### 2.3. Search Strategy 

The MEDLINE (via PubMed), ProQuest and Scopus databases were used to search for the three subject areas to be studied between October 2022 and February 2023 since, given the volume of the literature explored, an adequate saturation of information was achieved using this time range.

Search criteria were also used for the timely detection of information of interest in grey literature. The strategy comprised keywords for each disease combined with terms referring to epidemiological factors such as incidence, prevalence or risk factors, and in turn, the keywords were combined with the Boolean operators AND and OR. Each strategy can be seen in (Table A1). 

### 2.4. Study Selection and Data Extraction

**Figure 1:**
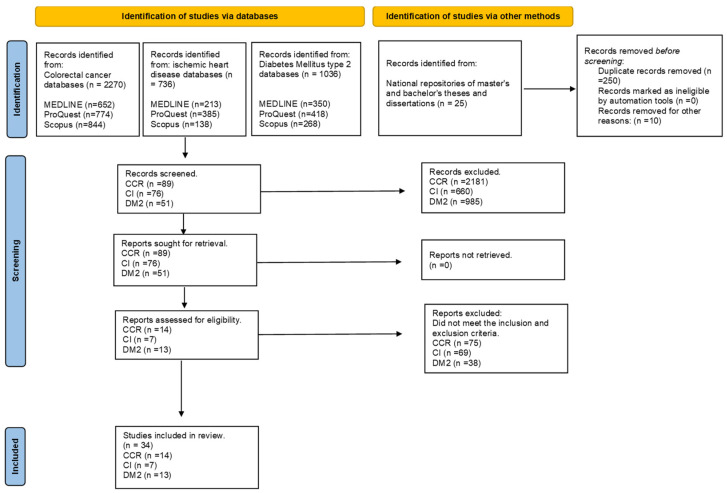
PRISMA statement flow diagram.

After applying the search strategy for each disease, the reviewers reached a consensus on how to approach data selection and extraction. One reviewer proceeded to assess the titles and abstracts of the studies found. The selected articles were then read through and assessed for inclusion and exclusion criteria by two reviewers independently. Once the criteria had been applied, the third reviewer verified the duplicate articles and proceeded to eliminate them. The articles to be analysed were selected and the team of reviewers prepared a table with the main characteristics for each article, for example, author’s name, year of publication, region of Spain, objective, methodology used and the relevant results of the study. Once the table was prepared, the reviewers independently assessed the methodological quality of the studies. Finally, a total of 34 articles, which covered the three diseases, were included, comprising 14 articles on colorectal cancer, 7 on ischaemic heart disease and 13 on diabetes mellitus type 2. The selected articles and their extractions are presented in the PRISMA flow chart (Figure 1).

### 2.5. Methodological Quality Assessment and Risk of Bias

The STROBE scale, which consists of 22 items that are converted into items for the assessment of the title, introduction, method, result and discussion of articles, was used to analyse the selected observational studies [25]. For experimental and clinical trial studies, the PEDro scale, made up of 11 items that assess the external and internal validity of studies, was applied [26]. The COREQ scale [27], the instrument for critical reading and evaluation of cross-sectional epidemiological studies, was also used for qualitative studies and for epidemiological research [28]. For the risk of bias, shown in Table 1, we used the Cochrane Handbook with the RoB 2 tool for randomised trials, where we assessed the different domains: bias arising from the randomisation process, bias due to deviations from the intended interventions, bias due to missing outcome data, bias in outcome measurements and bias in the selection of the reported outcomes [29]. Another tool used was ROBINS-I, which was used for non-randomised studies. The bias domains included are bias due to confounding, bias in the selection of study participants, bias in the classification of interventions, bias due to deviations from intended interventions, bias due to missing data, bias in the measurement of outcomes, and bias in the selection of reported outcomes [30]. The tools were managed using Review Manager software, version 5.4.1.

### 2.6. Data Analysis

A mixed-study narrative analysis was used in this study. All researchers read the selected articles thoroughly. After data extraction, a search for the most relevant results of each study was performed; themes of interest and key aspects for each disease were identified and then described in narrative form according to the inclusion criteria of the review.

## 3. Results

After a comprehensive database search, we found a total of 2.270 publications on CRC, 736 on IC and 1.036 on DM2. After eliminating duplicates and applying inclusion and exclusion criteria, 89 articles related to CRC, 76 to IC and 51 to DM2 were reviewed. Finally, fourteen studies on CRC, seven on IC and thirteen on DM2 were included in the review. The characteristics of the studies are shown in Table 1.

In the synthesis of each disease, the main findings of each study are presented in narrative form, highlighting the most relevant issues. 

### 3.1. Colorectal Cancer

In Spain, CRC accounts for 15% of all tumours diagnosed, with more than 25,000 new cases each year, making it the second leading cause of cancer deaths, with more than 13,000 deaths annually [65].

#### 3.1.1. Implementation of Screening and Early Detection Tests

The implementation of screening has led to positive changes in the Spanish population. In recent years, screening and detection tests have helped in the early detection of the disease. According to Zamorano-Leon et al., in their 2020 [39] study on the uptake of FOBT (faecal occult blood test) programmes for CRC, it was reported that those regions participating before 2011 (Canarias, Cantabria, Castilla y León, Cataluña, Comunidad Valenciana, País Vasco and La Rioja) showed a 3.38-fold increase in uptake over time, while for regions within the period 2011 to 2016 (Andalucía, Aragón, Asturias, Baleares, Castilla de la Mancha, Galicia and Navarra) the uptake increased from 4.3% to 13.2%. In contrast, those regions that did not run these programmes or were incomplete after 2016 (Extremadura, Madrid, and Ceuta and Melilla) also displayed an increase in acceptance from 3.4% to 8.8%. Following this analysis, and in general terms, it is remarkable that the Spanish population from the period 2011 to 2017 demonstrated such a positive acceptance of FOBT programmes. Another study, conducted in 2017 to measure the coverage of CRC screening in a sample of people aged between 50–69 years, showed that the communities with the highest levels of participation for an FOBT programme were the Basque Country (72.3%), Navarra (60.5%) and Castilla y León (49.1%); on the contrary, low participation was evidenced in Extremadura (8.7%), Ceuta and Melilla (10.4%) and Andalucía (14.1%) [33]. The study by Perestelo-Perez et al. (2019) [40] revealed the importance of information and knowledge that patients are provided with by primary care providers about existing screening tests. This study found that a part of the sample obtained a significant result for information issues, while another part showed no significance as they had prior knowledge of the tests. The majority of the participants reported that they underwent an FOBT (92.4%) or a colonoscopy (89.5%).

A study conducted in Aragon, with the aim of describing the first results of the CRC screening programme in this region, returned a participation rate of 45.28%, 95% CI (44.41–46.15). Of the overall rate, 10.75% of patients returned a positive FOBT, with a higher rate for men than women (13.77% vs. 7.92%); 95.07% of these participants then agreed to undergo colonoscopy. Lesions detected and the percentage of cancers by stage were also evaluated. Notably, these indicators showed that the Aragon region obtained acceptable values in the study [41]. Another analysis showed the importance of following an intervention protocol in collaboration with a multidisciplinary team in the search for better outcomes and survival for patients suffering from CRC. In this study, the intervention applied reached 72.41%, which is a high value for the efficiency and efficacy applied to participants who are screened [42]. 

#### 3.1.2. Determinants in Screening

In the studies analysed, certain health determinants that may affect the uptake of screening were detected. In their analysis of rural and urban areas in the province of Cuenca, González et al. (2022) [31] reported findings that showed the highest participation was in rural regions. It was also found that the social determinants of screening and early diagnosis can have a heterogeneous influence on the accessibility of the population at the time. For example, place of residence, annual income and unemployment rate were associated with the presence of adenomas and adenocarcinoma. Other biological and lifestyle factors that could be influential include age, foreign birth, illiteracy, active smoking, people who do not consume fruits and vegetables, self-perceived good health and those who make little use of health services [33].

#### 3.1.3. Healthy Lifestyle and Genetics in CRC

A number of studies detected factors that have an impact on healthy lifestyle and family history in relation to CRC [32,36,38]. In their population-based study in the Basque Country, Alegria-Lertxundi et al. (2020) [32] stated that the presence of diagnosed CRC occurred more in men than in women (4.8% vs. 2.1%) and that the interaction of genetic factors with healthy lifestyle plays an important role in decreasing the risk of CRC. In agreement with Solís-Ibinagagoitia et al. (2020) [38], they found that men were more prone to risk factors such as smoking, obesity, hypertension and diabetes than women. Also, men were less likely to use primary care services (45.4% men vs. 52.5% women). Similarly, the case group in the study by Rubín-García et al. (2022) [36] comprised males with a higher presence of high BMI levels, sedentary lifestyle, alcohol consumption, low educational levels and with a higher percentage of first-degree family history. Another important point of their research was the relationship between first-degree family history and age for the risk of CRC. In line with this study, a greater probability of developing the disease before the age of 50 was found. Similarly, the cohort study by Perea et al. (2021) [34] found that diagnosis was more prevalent between the ages of 40 and 49; it is worth mentioning that 42.6% of their participants were obese and 31.3% of those with a family history of cancer had a positive history of CRC. This study showed that the disease appears at an early age. Another study showed that a family history of CRC was the most important individual risk factor and that modifiable risk factors were stronger predictors of risk than genetic susceptibility [44]. 

#### 3.1.4. Mortality

Three studies addressed mortality in general and hospital mortality. In their study, Darbà and Marsà (2020) [35] aimed to update data on disease incidence and mortality in primary care areas and hospitalisations in Spain between 2011 and 2016. The lowest hospital mortality rates were in the regions of Cantabria, Catalonia and La Rioja, and the highest in the regions of Ceuta and Melilla, and the Canary Islands. The incidence of CRC in the hospitalised population was 106.39 per 10,000 patients for the years between 2011 and 2015. However, in 2016, this incidence dropped to 88.05 per 10,000 patients. It is worth noting that mortality increased with the age of the patients, specifically, 7.10% in those under 71 years old and 15.99% in those over 80 years old. Another study found that in Spain, CRC mortality was increased in both men and women. Males experienced an increase from 2256 new cases in 1980 to 9222 in 2018, while for females the number of cases increased from 2285 to 6066 in absolute values for the same period. In males, upward trends were recorded in almost all autonomous communities, two examples being Castilla–La Mancha (2.4%, *p* < 0.05) and Extremadura (2.2%, *p* < 0.05), with only the Basque Country remaining stable [37].

In relation to hospital mortality in Spain during the period 2008–2014, 258,927 episodes of hospitalisation were evaluated, with an average patient hospital stay of 13.16 days, the majority of whom were male (60.6%). As for the number of hospital admissions for CRC, in those patients older than 20 years, the study showed an increase from 34,111 cases in 2008 (crude rate 73.90/100,000 population) to 38,591 in 2014 (crude rate 82.51/100,000 population). Finally, the national average annual CRC mortality rate was 20.0 per 100,000 population. Following this analysis, an increase in rates in the regions of Galicia, Asturias, Cantabria, Basque Country, La Rioja, Castilla León, Extremadura, Valencia and Catalonia was observed [43]. 

### 3.2. Ischaemic Heart Disease

#### 3.2.1. Risk Factors Associated with Ischaemic Heart Disease

Most of the studies analysed referred to risk factors for IHD in Spain. In their study conducted in the ICU of La Ribera hospital, Estarlich et al. (2022) [45] showed that the individual risk factors associated with the severity of ischaemic heart disease were age, previous IHD and dyslipidaemia. Another study conducted in a public administration office in Cordoba showed that age, blood pressure and smoking were the risk factors most associated with cardiovascular events (IHD and stroke) [48]. Similar results were reported by Mendoza Alarcón et al. (2021) [49] in Badajoz, who, supported by the JARA system of the Extremadura Health Service, showed that the risk factors associated with the disease were as follows: 77.9% for hypertension, 69.3% for dyslipidaemia, 48.2% for obesity and 32.3% for diabetes. Furthermore, Melero-Alegria et al. (2019) [51], in their analysis of the SALMANTICOR study, assessed structural heart disease stratified by age, sex and place of residence, in addition to assessing the risk factors of their participants. Meanwhile, a study by the Spanish Society of Cardiology for two registries between 2006 and 2014 showed that over the course of the study period, the prevalence of DM (diabetes mellitus) and AHT (arterial hypertension) decreased, even though the presence of smokers increased. Notably, there were improvements in dyslipidaemia, resting heart rate, and blood glucose in diabetics and non-diabetics. In contrast, obesity control did not show improvements in the study [50].

#### 3.2.2. Types of IHD

These studies revealed the types of ischaemic heart disease with the greatest impact on health. In view of the above, one study established that more than half of the participants (66.4%) had an episode of acute inferior myocardial infarction (40.1%) [45]. In line with this, in another study conducted in Navarra with 12.8 years of follow-up, the RIVANA cohort showed 194 important points related to cardiovascular situations such as combination of AMI, stroke or death due to stroke as primary study events. Secondary criteria were myocardial infarction, stroke, cardiovascular problems and complex cerebrovascular situations [46].

#### 3.2.3. Healthy Lifestyle

Fernandez-Lazaro et al. (2022) [46], in their study on the RIVANA cohort, set out an analysis of Life’s Simple 7 metrics with the AHA’s (The American Heart Association) ideal cardiovascular health concept (ICVH) for improving health and reducing deaths from cerebrovascular disease. Of the total participants, 90.6% followed the physical activity metric as a whole, and for each individual metric: 64.4% smoking, 60.2% glucose, 56.2% healthy diet, 26.4% blood pressure, 36.4% BMI and 33.6% cholesterol. Another study highlighted how 21.7% of its participants achieved positive results in therapy, i.e., no smoking, taking medication and a reduction in LDL cholesterol levels [49].

#### 3.2.4. Mortality and Incidence

In their study on premature mortality due to IHD in Spain during the period 1998–2018, Hervella et al. (2021) [47] found a total of 232,617 premature deaths, of which 78% were males and 22% were females. In terms of data, the figure for premature mortality recorded in 1998 was 14,876 people, while in 2018 this figure had dropped to 8780 people, with females exhibiting the lowest percentage. The age range with the highest mortality was people over 74 years of age. In terms of geographical distribution, men displayed higher rates in Andalusia, Valencia, non-peninsular communities, Extremadura, Murcia, Asturias and Lugo. Conversely, in women, the most unfavourable rates were found in the southern and eastern regions, Melilla, Asturias, Coruña, Soria and Cuenca.

Only one article referred to incidence. This study was carried out in Cordoba, with a sample of 698 workers from a public administration office. The incidence of ischaemic heart disease was 276.3 per 100,000 person-years and for cardiovascular disease (IHD and stroke), it was 360.1 per 100,000 workers per year [48].

### 3.3. Diabetes Mellitus Type 2

#### 3.3.1. Risk Factors and Incidence of Diabetes Mellitus Type 2 (DM2) in Spain

In their population-based study “Di@bet.es”, a prospective cohort implemented by the Spanish Diabetes Society, Martínez-Hervás et al. (2022) [52] detected higher baseline age, BMI, waist circumference, blood pressure, FPG (fasting plasma glucose), HOMA (Homeostatic Model Assessment), HbA1c, LDL and FTG (fasting triglycerides), and lower HDL as risk factors in their participants after a follow-up of 7.5 years. In addition, the study showed a significant association for FPG, age and FTG as a risk for developing DM2. In connection with the above, Hawkins Carranza et al. (2022) [53] showed in their analysis that the predictor risk factors in their study were BMI, hypertension, occupation and the incidence of DM2. Another study carried out in Tenerife by Cuevas Fernández et al. (2021) [54] highlighted that their participants displayed obesity, low educational level, dyslipidaemia, hypertension and metabolic syndrome as risk factors.

Regarding incidence, Martínez-Hervás et al. (2022) [52] found that after 7.5 years of follow-up, those patients who developed DM2 had a crude incidence of 8.5 per 1000 person-years. Also, another study in people over 65 years of age in three central areas of Spain (Las Margaritas, Lista and Arevalo) showed a mean incidence of type 2 DM of 9.8 per 1000 person-years. In these studied communities, the incidence was highest in Margarita (11.36), followed by Arevalo (10.14) and Lista (7.6) [53]. Only one study made reference to the fact that the higher the weighting of the “healthy lifestyle” factor, the lower the risk of incidence of type 2 DM [56]. 

#### 3.3.2. Preventive Measures Applied for the Control of the Disease

The following studies applied measures and analyses that facilitated the necessary strategies or measures for disease prevention. Among them is the study by López-Cobo et al. (2022) [64] in Barcelona, that compared metabolic control and vascular risk factors. The results showed adequate glycaemic control between 2012 and 2016. In addition, improvements in LDL cholesterol and HT levels were observed. Another article with an intervention method applied to a population of adolescents with weight problems in the Basque Country showed significant differences in time trends for BMI, favouring the intervention group (IG). Also, in terms of physical activity, significant differences were observed between those participants who dedicated an hour of their time to exercise and those who did not. Another important finding for the IG was an increase in fruit and vegetable consumption and improvements in eating habits [55]. Ruiz-Estigarribia et al. (2020) [56], in their individual analysis of healthy lifestyle components (HLS), showed that never smoking, moderate to high physical activity and a moderate to high intake of a Mediterranean diet were factors that helped with diabetes risk. Similarly, the results of Represas-Carrera et al. (2021) [63] showed a marked improvement in adherence to the Mediterranean diet, with the intervention group delivering the best results. In the analysis applied by Martin-Ridaura et al. (2022) [58], after a pre-post intervention evaluation of the ALAS programme, which aims to promote healthier lifestyles, reduce obesity and prevent the development of type 2 diabetes in the population of Madrid, remarkable results were obtained. In the first instance, 6 months after the intervention, participants showed a decrease in their initial weight, waist circumference and BMI. At 12 months, both weight and waist circumference had decreased further. In their study, Martos-Cabrera et al. (2021) [61] noted that after the intervention, HbA1c levels were reduced in both groups. The most favourable group in this study was the intervention group, as they were able to reduce the levels of glycosylated haemoglobin.

Another qualitative study of a multidisciplinary group of physicians in Spain expressed the importance of early screening in patients with type 2 DM, healthy lifestyle interventions, glycosylated blood glucose testing and glycaemic control as key strategies for reducing the prevalence of the disease [60].

In contrast, two studies, one descriptive and one RCT, showed evidence of poor diabetes control. Cuevas Fernandez et al. (2021) [54] found that 24% of their participants had poor diabetes control and 25% had diabetes complications. This poor management was reflected in males under 65 years of age. These patients had poor triglyceride, HDL and triglyceride/HDL ratios. In addition, they had an inadequate diet, poor therapeutic management and no ECG (electrocardiogram). Another result showed non-significance with glycaemic control, physical activity, sedentary lifestyle, smoking and quality of life during the study period [63]. 

#### 3.3.3. Empowering the Patient with Type 2 DM

A study on patient empowerment conducted in four of the Canary Islands (Tenerife, Gran Canaria, Lanzarote and La Palma) established that the factors significantly associated with empowerment in participants were sociodemographic and clinical factors, age, gender, educational level, living alone, employment status, country of birth, time since diagnosis, HbA1c and number of comorbidities. Other factors such as knowledge of diabetes, anxiety status, depression, distress and diabetes-related quality of life were also key for patient empowerment [59]. In their study, Vilafranca Cartagena et al. (2022) [62] demonstrated the importance of the knowledge and empowerment that patients should have when dealing with the disease; for example, their participants had to learn to cope with the disease and acquire new knowledge. In addition, participants expressed agreement that PA (physical activity) is important in improving the disease, along with diet, hereditary factors and drug treatment, which also played a role in the diagnosis. 

#### 3.3.4. Comorbidities in Patients with DM2

Using the databases of the Information System for the Development of Primary Care Research (SIDIAP) in Catalonia, Mata-Cases et al. (2019) [57] reported that patients over 75 years of age showed greater comorbidity than younger patients. The most frequently reported comorbidities were hypertension (72%), hyperlipidaemia (60%), obesity (45%), chronic kidney disease (33%), chronic renal failure (28%) and cardiovascular disease (23%). Also, López-Cobo et al. (2022) [64] stated that the notable chronic complications of type 2 DM in their study were diabetic retinopathy, diabetic neuropathy (subjects having type 2 DM for at least 10 years), heart failure and peripheral vascular disease.

## 4. Discussion

This review describes three important non-communicable diseases (CRC, IHD and DM2) which, due to their clinical relevance and prevalence, represent a public health problem. Specifically, this review allowed us to evaluate the current situation in Spain in relation to these three diseases. For this reason, our study analysed a total of thirty-four articles, fourteen corresponding to colorectal cancer, seven to ischaemic heart disease and thirteen to type 2 diabetes mellitus. In general, these studies revealed the most prevalent risk factors in Spain, as well as the most appropriate preventive measures and types of healthy lifestyle for each of them. Regarding healthy lifestyle, responsible human behaviour has played a major role in the analysis of these three diseases. However, few studies have dealt with mortality, incidence and prevalence data.

In the case of colorectal cancer, four key issues stand out, namely, implementation of screening and early detection tests, determinants of screening, healthy lifestyle and genetics, and mortality. With regard to screening and early detection, mention is made of people’s positive acceptance of FOBT programmes [39]. In addition, screening coverage [33], information and awareness of the existence of the tests [40], participation rates [41] and having an intervention protocol in place [42] were key points when screening for the disease. However, a cross-sectional study by the Spanish National Health Survey (ENSE17) shows low participation in terms of screening coverage in the regions of Extremadura, Ceuta and Melilla, and Andalusia. In contrast, the regions of the Basque Country, Navarra and Castilla y León showed higher participation [33]. These data are in agreement with a study conducted in Nebraska, where Gonzales et al. (2023) [66] stated that CRC screening in conjunction with education are important points in the detection of the disease. Recently, a study in Spain concluded that the uptake of FIT (faecal immunochemical testing) between 2017 and 2020 increased, but the prevalence of FIT remains low compared to European guidelines [67].

Additionally, there are determinants that can influence the screening process. For González et al. (2022) [31] and Nouni-García et al. (2022) [33], these factors, in particular, are place of residence, economic and employment status, biological factors and healthy lifestyle. In addition to poor practice of good health habits, family history with CRC represented a core risk factor for developing the disease [32,34,36,44]. Similarly, Portero de la Cruz and Cebrino (2023) [67] found that socioeconomic status, level of education, social status and nationality were factors that intervened in the detection process. Also, a study conducted in a rural area demonstrated the needs for practitioners and the challenges that the population are faced with regarding the limitations of CRC screening [68]. Two studies reported the presence of the disease before the age of 50 years [34,36]. The appearance of the disease at younger ages is worrying when the evidence has shown that it mostly appears after the age of 50 years; in fact, the American Cancer Society (ACS) has reduced the screening pattern for CRC to 45 years due to the increased incidence in populations under 50 years of age [66]. In accordance with the above-mentioned factors, it is necessary to highlight the short- and long-term complications of the pathology itself. For CRC, we can highlight the prevalence of obesity, mental disorders, metastasis and liver involvement. This is consistent with studies showing that obese men have a higher risk of CRC than obese women [69]. Also, Popovici et al. (2023) [70] provides evidence for a significant association between obesity and CRC. On the other hand, Cheng et al. (2022) [71] highlights the association between depressive disorders and patients with CRC. Another complication is the presence of metastases, with the liver region being the most affected. This is in agreement with Chávez-Villa et al. (2023) [72], who detail among their findings the presence of occult carcinomas, specifically in colorectal liver metastases. In terms of mortality and incidence, only three studies of those analysed made reference to this. In one of the articles aiming to update the data between 2011 and 2016, low rates of hospital mortality occurred in the regions of Cantabria, Catalonia and La Rioja, while high rates occurred in the regions of Ceuta and Melilla, and the Canary Islands. It is worth mentioning that the incidence until 2016 had dropped to 88.05 per 10,000 patients [35]. In contrast, by 2014 these rates had increased in the regions of Galicia, Asturias, Cantabria, Basque Country, La Rioja, Castilla León, Extremadura, Valencia and Catalonia [43]. Additionally, in their study up to 2018, Cayuela et al. (2021) [37] showed that the Spanish population experienced an increase in mortality for both men and women. 

For ischaemic heart disease (IHD), the risk factors most associated with the disease were age, HTA, dyslipidemia, smoking, obesity and diabetes [45,48,49,50]. These results are consistent with several studies, for example, Avan et al. (2023) [73] and Dai et al. (2022) [74], who indicate that modifiable risk factors such as HTN, cholesterol, air pollution, alcoholism, smoking and unhealthy diets are related to IHD, while Liu et al. (2022) [75] points out that depression and obesity were the main risk factors in their analysis. Accordingly, our findings indicate that the types of ischaemic heart disease with the greatest impact were acute myocardial infarction and a combination of AMI with stroke or cardiovascular problems [45,46]. In relation to this, there is evidence of complications in which ischaemic heart disease is related. For example, in their study in France, Lecoeur et al. (2023) [76] reported that heart failure in relation to ischaemic heart disease is affecting younger patients every day. Another study in India showed that heart disease and angina were more representative in the population over 70 years of age [77]. 

As per healthy lifestyle, Fernandez-Lazaro et al. (2022) [46] and Mendoza Alarcón et al. (2021) [49] reported that lifestyle was focused on participation in physical activity, reduction of smoking, diabetes and cholesterol, control of HTN and a healthy diet. These results are consistent with studies where healthy practices were associated with reduced development of cerebrovascular disease, including ischaemic heart disease [78,79]. Regarding mortality, there are few studies with up-to-date data on the subject. However, Hervella et al. (2021) [47] analysed premature mortality due to IHD in Spain between 1998–2018. They found that this mortality had decreased by 2018, with women having the lowest percentage. Following this study, the highest rates in men were in Andalusia, the Valencian Community, extra-peninsular communities, Extremadura, Murcia, Asturias and Lugo. And, in women, the most unfavourable rates were found in the southern and eastern regions, Melilla, Asturias, Coruña, Soria and Cuenca. In terms of the incidence rate of workers, only one study emphasised this, in a public administration office in Cordoba, with figures of 276.3 per 100,000 person-years [48]. This information aligns with Fernández-Bergés et al. (2022) [80], who found that there is little scientific evidence to report on ischaemic heart disease in Spain. Furthermore, Lazarus et al. (2022) [81] indicated in their analysis of the GBD study (Global Burden of Disease Study) 2019 that IHD and CA HNCDs contribute to mortality and morbidity in Spain. 

Finally, with regard to type 2 diabetes mellitus (DM2), four themes emerged in our results: risk factors and incidence of diabetes in Spain, preventive measures applied to control the disease, patient empowerment and comorbidities in patients with DM2. Risk factors included older age at baseline, BMI, waist circumference, AHT, abnormal values in blood tests (HbA1c, LDL, HDL, triglycerides, FPG, HOMA), occupation, obesity, low educational level and metabolic syndrome [52,53,54]. These data are in agreement with other studies establishing that increased BMI, smoking, environmental pollution, being overweight and below normal waist circumference values act as risk factors [82,83]. Regarding incidence, only three studies made reference to this. The first one, with 7.5 years of follow-up performed on the Spanish cohort, was Di@bet.es, which showed that its participants had an incidence of 8.5 per 1000 person-years [52]. The second, which applied to a population of people over 65 years old in three central areas of Spain (Las Margaritas, Lista and Arévalo), found an incidence of 9.8 per 1000 person-years [53]. The third one, in Navarra, showed that the healthier the lifestyle, the lower the incidence of T2DM [56].

Preventive measures applied to the disease are of great benefit to diabetic patients. These include maintaining adequate metabolic and risk factor control, physical activity, consumption of fruit and vegetables with improvements in eating habits, avoidance of smoking, reduction in HbA1c levels, weight and BMI reduction, which are all part of the basis of guidelines that help to reduce glucose levels [55,56,58,61,64]. These findings coincide with other studies such as that of G. Liu et al. (2023) [84] and another conducted in China by Wu et al. (2022) [85], in which they establish that leading a healthy lifestyle (healthy diet, not smoking, exercise, and the consumption of vegetables and fruit) reduces the risk of complications and becomes a protective factor against DM2. In addition, two studies demonstrated the benefits of the Mediterranean diet for diabetic patients [56,63]. Another point of interest is the patient–doctor relationship, and accordingly, a qualitative study by a group of doctors in Spain demonstrated the importance of early screening in patients with T2DM, in tandem with good practices, disease control and dietary habits [60]. Conversely, poor diabetes control and poor healthy lifestyle practices lead to complications of the disease [54,63].

With regard to patient empowerment, and following the evidence presented, there are factors that need to affect the patient in order for them to become empowered by their disease. These factors are sociodemographic and clinical: age, gender, educational level, living alone, employment status, country of birth, time since diagnosis, HbA1c, number of comorbidities, knowledge of the disease, emotional state and quality of life [59,62]. Similarly, in a study conducted in Pakistan, Khowaja et al. (2023) [86] highlighted among their results that there are predictors for the empowerment of patients with DM, placing emphasis on knowledge of the disease, treatment, foot care, age, marital status and economic status. There are another two studies in our review that reveal that diabetic patients, in addition to suffering from the disease, also experience certain comorbidities and complications such as hypertension, hyperlipidaemia, obesity, chronic kidney disease, chronic renal failure, cardiovascular disease, diabetic retinopathy, diabetic neuropathy, heart failure and peripheral vascular disease [57,64]. This coincides with the analyses of Dimore et al. (2023) [87] and Luo et al. (2023) [88], which highlight complications in patients with DM2 including retinopathy, foot ulcers, nephropathy and neuropathy. Similarly, Berhe et al. (2023) [89] identified within their analysis microvascular complications such as eye disease, renal disease and peripheral neuropathy, as well as macrovascular complications, with hypertension being the most common and cerebrovascular disease the least common. 

### Strengths and Limitations

The strength of this study is the analysis of three major diseases with a high prevalence in Spain. Through an in-depth and thorough review of each disease, it was possible to extract and analyse both relevant issues and the presence of risk factors shared by each of them. In addition, they were considered as pathologies that have an impact on the increase in mortality in the Spanish population. Another strength lies in the methodological assessment applied to each study, carried out in collaboration with tools that allow us to identify bias in the publications. This allowed us to provide high-quality information and a critical perspective on the most recent developments in relation to the three diseases. To date, there are no studies that have addressed these three major pathologies together. This analysis will help to identify the current situation and the public health measures that have been implemented to date. 

Our review had some limitations. Firstly, the heterogeneity of the reviewed studies was a limited factor, as we included observational, epidemiological, experimental and qualitative studies. This made it difficult to combine results and draw solid conclusions. In addition, we included studies based on national registries, which makes them vulnerable to bias. We also identified four articles with unclear risk of bias, one with high bias and two of qualitative design through methodological quality, which was not applicable due to the scientific difficulty of measuring bias in this type of study. Another limitation was the small final sample of studies related to ischaemic heart disease, so that selective publication could be activated at the time of article selection. This may be related to the fact that in recent years there has been a certain bias derived from the attention being drawn away by COVID-19, temporarily diminishing interest in chronic diseases. For all these reasons, further studies related to the three diseases are recommended, as well as making updated statistical analyses publicly available, ensuring that the Spanish population has the information required to earn their confidence. Moreover, further detailed analysis of the cardiovascular risk factors that, for the most part, underlie the three major entities studied should be continued in future studies. Diabetes itself is such an important entity that it has been included in the initial study; however, many other risk factors should be the target of future studies and reviews.

## 5. Conclusions

Ischaemic heart disease, type 2 diabetes mellitus and colorectal cancer, as chronic non-communicable diseases, have become significant causes of death in Spain. It is notable that risk factors and lifestyle have an impact on the incidence, prevalence and mortality of the diseases studied and analysed. As such, the responsible behaviour of human beings, associated with their lifestyle, is directly related to the occurrence of these three diseases. 

## Figures and Tables

**Table 1:** Characteristics of the studies.

Author and Year	Disease	Region of Spain	Study Design	Risk of Bias	Participants and Population	Variables	Results
González et al., 2022 [31]	CCR	Province of Cuenca	Retrospective, descriptive, analytical and observational study	Low	n = 1422 male and female patients between 50 and 69 years old	Sociodemographic, economic and clinical data	Present determining factors of screening: place of residence, annual income and unemployment rate.
Alegria-Lertxundi et al., 2020 [32]	CCR	Osakidetza/Basque Service	Observational study epidemiological study	Low	n = 308 cases diagnosed with CRC and n = 308 controls. Participants (between 50 and 69 years old)	The evaluation of dietary intake, lifestyle, demographic and socioeconomic determinants, and genetic factors.	The presence of diagnosed CRC occurred more in men than in women (4.8% versus 2.1%). The interaction of genetic factors with a healthy lifestyle plays an important role in reducing the risk of CRC.
Nouni-García et al., 2022 [33]	CCR	All of Spain	Transversal study	Low	n = 10,595 men and n = 12,494 women. Men and women between 50 and 69 years old.	Faecal occult blood (FOBT). On the other hand, the explanatory variables covered sociodemographic variables, health determinants, medical care variables and self-perceived health.	The communities with the most participation that had had an FOBT were the Basque Country (72.3%), Navarra (60.5%) and Castilla y León (49.1%). On the contrary, low participation was represented by Extremadura (8.7%, n = 16), Ceuta and Melilla (10.4%, n = 3) and Andalusia (14.1%, n = 186).
Perea et al., 2021 [34]	CCR	All of Spain	Multicentre prospective cohort	Unclear	n = 220 patients with early onset colorectal cancer Investigation (EOCRC)	Demographic, clinic-pathological characteristics of EOCRC and molecular characterisation.	60.3% of the cases were men and the average age was 44 years. Regarding location, the tumour with the most cases was the rectum (42.6%), then the left colon (32.4%) and the right colon (25%).
Darbà and Marsà, 2020 [35]	CCR	All of Spain	Retrospective multicentre observational study	Low	n = 99,653 income records	The variables studied include the information recorded from the patient’s profile and details of the admission.	The male sex obtained the highest percentage both in the records obtained from primary care (56.17% men and 43.83% women) and those from hospitalised care (60.70%). The lowest hospital mortality rates by regions occurred in Cantabria, Catalonia and La Rioja, and the highest in Ceuta and Melilla, and the Canary Islands.
Rubín-García et al., 2022 [36]	CCR	Leon, Barcelona, Madrid, Asturias, Cantabria, Guipuzcoa and Navarra, Granada, Huelva, Murcia and Valencia	Observational, multicentre, multicase-control study	Low	Cases, n = 1360 and controls, n = 2857. The participants ranged in age from 20 to 85 years.	The variables to be studied: information on diet, sociodemographic data, anthropometric data, environmental exposures and lifestyles, and physical activity.	The cases were higher than the controls. In this case, the presence of first-degree FA (family history) increased 4 times (aOR: 4.22; 95% CI: 2.29–7.78) and with a family member diagnosed before the age of 50 there were three times more likely (aOR: 3.24; 95% CI: 1.52–6.91).
Cayuela et al., 2021 [37]	CCR	All of Spain	Observational record	High	CRC death records (1980–2018).	Age-standardised mortality rates for CRC. For these rates, individual records broken down by sex, age and year of death were used.	Mortality from CRC increased in both men and women. Men experienced an increase from 2256 new cases in 1980 to 9222 in 2018, while women increased the number of cases from 2285 to 6066 in absolute values for the same period.
Solís-Ibinagagoitia et al., 2020 [38]	CCR	Basque Country	Study was cross-sectional descriptive	Low	n = 515,388 people. Target population between 50 and 69 years old	Age, sex, smoker, diabetes, high blood pressure, obesity, use of health services, vaccination, comorbidity, deprivation, type of participants. Non-participation was a fundamental aspect of the study.	It was observed that men were more prone to risk factors such as smoking, obesity, hypertension and diabetes than women. On the other hand, 45.4% of men use APS services, less than women (52.5%).
Zamorano-Leon et al., 2020 [39]	CCR	All of Spain	Transversal study	Low	n = 12,657. Participants aged 50 to 69 years old	Adoption of FOBT-based CRC screening, sociodemographic characteristics, health status, and lifestyle behavior.	The Spanish population from 2011 to 2017 had a positive acceptance of FOBT programs.
Perestelo-Perez et al., 2019 [40]	CCR	Tenerife	Randomised controlled trial study	Low	n = 107 patients, of which n = 83 belonged to centre A and n = 24 to centre B.	Decision aid (DA), decisional conflict, knowledge about CRC and screening options	The effect of the intervention on the decisional conflict showed that centre A had better acceptance of the intervention than centre B. For its part, centre A obtained a beneficial effect on the decision-making process, favouring the intervention group.
Solé Llop et al., 2018 [41]	CCR	Aragon	Prospective observational	Unclear	n = 12,518 people, population aged 60–69 years	The screening process indicators were evaluated: indicators of detected injuries; indicators of detected tumours; and positive predictive value.	Of the overall rate, 10.75% of patients were positive for the FOBT test, and was higher in men than in women (13.77% vs. 7.92%). Regarding the colonoscopy, 95.07% of the participants agreed to have it done.
Álvarez-delgado et al., 2021 [42]	CCR	Salamanca	Experimental or intervention study	Low	n = 33,167 patients aged 60 to 69 years	Sex, comorbidities, use of anticoagulants, allergies, patient’s initiative to participate, patient preparation, food intake before the intervention, abstinence time, tolerance to the preparation, intestinal cleansing with the scale of Boston, faecal intubation, divided dosing, clinical-preventive relevance, efficacy of colonoscopy as treatment and location of adenomas.	Regarding the characteristics of the population, there were no significant differences in age, sex, comorbidities, the use of antiplatelet drugs, use of anticoagulants or allergies between the intervention and control groups. Both the IG and the CG were similar and homogeneous, and therefore comparable.
García-Torrecillas et al., 2019 [43]	CCR	All of Spain	Cohort study	Low	n = 258,927 hospitalisation episodes	The main variable analysed was mortality during hospitalisation. Other variables studied were sociodemographic, clinical features such as discharge diagnoses and discharge procedures. Other variables analysed were level of severity, type of admission and management variables.	258,927 hospitalisation episodes were evaluated. As for the average hospital stay, it was 13.16 days, with the majority being men (60.6%). The average annual CRC mortality rate nationwide was 20.0 per 100,000 inhabitants. An increase in rates was recorded in the regions of Galicia, Asturias, Cantabria, the Basque Country, La Rioja, Castilla León, Extremadura, Valencia and Catalonia
Ibáñez-Sanz et al., 2017 [44]	CCR	Madrid, Barcelona, Navarra, Girona, Gipuzkoa, Leon, Asturias, Murcia, Huelva, Cantabria, Valencia and Granada	Cases and controls study	Low	n = 1336 CRC cases and n = 2744 controls with genotype data. People between 20 and 85 years old	Variables to study: family history of CRC, smoking, BMI, average physical exercise, consumption of red meat and patient medications. For location anatomical distribution: proximal, distal colon and rectum.	Family history of CRC was the single most important risk factor for CRC and modifiable risk factors were stronger in predicting risk than genetic susceptibility.
Estarlich et al., 2022 [45]	IC	Alzira	Retrospective cross-sectional study	Low	n = 244 patients. Patients admitted to the ICU with a diagnosis of IC	Circadian rhythm variables, sociodemographic and risk factors, IHD severity variables, location of infarction and length of hospital stay.	The individual risk factors that were associated with the severity of ischaemic heart disease were age, previous IHD, and dyslipidemia.
Fernandez-Lazaro et al., 2022 [46]	IC	Navarra	Population cohort study	Low	n = 3826 people between 35 and 84 years old.	Sociodemographic and lifestyle data, medical history, medication use and biological parameters.	After the ideal metrics evaluated, a lower risk of major cardiovascular events such as AMI, stroke or deaths from cardiovascular causes were significantly associated.
Hervella et al., 2021 [47]	IC	All of Spain	Observational study	Low	n = 232,617 premature deaths due to IC. Ages between 0 to 74 years	The variables to be studied were age, sex, year, province of residence and year of death.	In data, premature mortality recorded in 1998 was 14,876 people, while in 2018 this figure was reduced to 8780 people, with the female sex having the lowest percentage.
Álvarez-Fernández et al., 2020 [48]	IC	Cordoba	Longitudinal cohort study	Low	n = 698 workers. Age between 35 and 60 years	Among the study variables were REGICOR’s own rating, whether or not to suffer from ischaemic heart disease, person and lifestyle, analytical and anthropometric variables.	Age, blood pressure and smoking were the risk factors most associated with cardiovascular events (IHD and stroke). The incidence for IHD was 276.3 per 100,000 person-years and for cardiovascular disease (IHD AND stroke) was 360.1 per 100,000 workers-year.
Mendoza Alarcón et al., 2021 [49]	IC	Bajadoz	Observational study	Low	n = 200 patients assigned to the La Paz health centre in Badajoz with hospital diagnosis	Prevalence of cardiovascular risk factors being hypercholesterolemia, tobacco consumption, high blood pressure, diabetes, obesity and chronic kidney disease. Also, their degree of control and drug adhesions.	This study revealed that the risk factors associated with the disease corresponded to 77.9% for high blood pressure, 69.3% for dyslipidemia, 48.2% for obesity and 32.3% for diabetes.
Cordero et al., 2016 [50]	IC	All of Spain	Data from two prospective, observational, multicentre registries	Unclear	In 2006, n = 1583 patients and in 2014, n = 1110 patients.	Among the variables to be studied was the record of all treatments and doses received before the visit and the optimal medical treatment (BMT). On the other hand, the control of HBP, heart rate, dyslipidemia, DM, obesity and glomerular filtration rate.	The study showed that diabetes (DM) and hypertension (HTN) decreased. Notable improvement in medical treatment, especially in control of risk factors.
Melero-Alegria et al., 2019 [51]	IC	Salamanca	Cross-sectional descriptive population-based study	Unclear	n = 2400 people over 18 years old	Medical history, different surveys including social status, Mediterranean diet, functional capacity, ECG, echocardiogram, VASERA, biochemical and genetic analysis.	Analysis of the SALMANTICOR study (study of the prevalence of structural heart disease and its risk factors). This study provides echocardiographic parameters.
Martínez-Hervás et al., 2022 [52]	DM2	All of Spain	Prospective, multicentre, population-based cohort study	Low	n = 2408 participants. People aged ≥18 years from the Di@bet.es study	Age, educational level, physical activity, smoking, dyslipidemia, medication and family history.	After a follow-up of 7.5 years, they detected as risk factors a higher initial age, BMI, waist circumference, blood pressure, FPG (fasting plasma glucose), HOMA (Homeostatic Model Assessment), HbA1c, LDL and FTG (fasting triglycerides), and lower HDL. The patients had a crude incidence of 8.5 per 1000 person-years.
Hawkins Carranza et al., 2022 [53]	DM2	Central Spain (Las Margaritas, Lista and Arevalo)	Cohort study	Low	n = 2000 people for each area. People over 65 years of age	Age, sex, educational level, occupation, physical activity (PA), weight and height, BMI and blood tests.	In their analysis, they show that the predictive risk factors in their study were BMI, hypertension, occupation and the incidence of T2D. In the three central areas of Spain studied (Las Margaritas, Lista, Arevalo), their results showed an average incidence of type 2 DM of 9.8 per 1000 person-years.
Cuevas Fernández et al., 2021 [54]	DM2	Tenerife	Retrospective cross-sectional descriptive study	Low	n = 587 patients with DM2	Good or bad control of T2DM according to the GDPS 2018 network. In addition, sociodemographic variables, habits, clinical variables, dyslipidemia, high blood pressure, metabolic syndrome, years of evolution of T2DM, ischaemic heart disease, complications of T2DM, general comorbidities and therapeutic guidelines.	There was a risk of obesity, low level of education, dyslipidemia, hypertension and metabolic syndrome. Of the total number of participants, 24% had poor diabetes control and 25% had complications of the disease.
Alustiza et al., 2021 [55]	DM2	Basque Country (Osakidetza)	Randomised controlled clinical trial	Low	n = 92 participants, n = 47 in the intervention group (IG) and n = 45 in the control group (CG); adolescents aged 12 to 14 years	Anthropometric measurements, vital signs, stage of pubertal development, diet, physical activity, sociodemographic characteristics, family, prenatal, personal history and laboratory parameters.	The increase in BMI was stopped through the intervention. Significant differences were observed between those participants who spent an hour of their time exercising and those who did not. There was an increase in the consumption of fruits and vegetables, and improvements in eating habits in the GI.
Ruiz-Estigarribia et al., 2020 [56]	DM2	Navarra	Prospective cohort study	Low	n = 11,005 participants, Spanish university graduates. Follow-up University of Navarra (SUN)	Sociodemographic aspects, lifestyle, anthropometric variables and medical history. Other variables to be studied were clinical variables such as prevalence of family history of diabetes, personal history of hypertension, hypertriglyceridemia and hypercholesterolemia status.	In their analysis of the healthy lifestyle (HLS) components, results showed that never smoking, moderate to high physical activity, and a moderate to high Mediterranean diet were factors that helped with diabetes risk.
Mata-Cases et al., 2019 [57]	DM2	Catalonia	Transversal study	Low	n = 373,185 patients, ≥18 years old with a diagnosis of DM2	Comorbidities, age, gender, smoking status, onset of DM2, date of DM2 diagnosis, blood pressure, cholesterol, weight, height, urine albumin creatinine to creatinine ratio (UACR) and glycated haemoglobin (HbA1c) and pharmacological treatments.	Patients over 75 years of age had greater comorbidity than younger patients. The most frequently presented comorbidities were hypertension (72%), hyperlipidemia (60%), obesity (45%), chronic kidney disease (33%), chronic kidney failure (28%) and cardiovascular disease (23%).
Martin-Ridaura et al., 2022 [58]	DM2	Madrid	Quasi-experimental study, pre-post evaluation of the intervention	Low	n = 1629 start of the intervention, n = 1021 end of the intervention; people between 35 and 69 years old	Weight, BMI, waist circumference and glycaemic levels. On the other hand, the sociodemographic variables, educational level, employment situation, origin and marital status.	Six months after the intervention, the participants showed a decrease in their initial weight, and a reduction in waist circumference and BMI. At 12 months, both weight and waist circumference decreased.
Duarte-Díaz et al., 2022 [59]	DM2	Canary Islands	Transversal study	Low	n = 2334 patients between 18 and 65 years old	The main variable was empowerment. Other variables to be studied were sociodemographic data, clinical data, knowledge of diabetes and quality of life related to diabetes.	Study that mentions patient empowerment. The factors that were significantly associated with empowerment in the participants were sociodemographic and clinical factors, age, sex, educational level, living alone, employment status, country of birth, time since diagnosis, HbA1c and the number of comorbidities.
Cos et al., 2022 [60]	DM2	Spain	Qualitative study	Low	n = 300 doctors primary care doctors and hospital care specialists	A Delphi-type survey was carried out with 25 statements and 13 questions on opinion, attitude and behavior (OAB).	Doctors expressed the importance of early screening for patients with type 2 DM, interventions for good healthy lifestyle practice, glycosylated testing and glycaemic control as key strategies to reduce the prevalence of the disease.
Martos-Cabrera et al., 2021 [61]	DM2	Andalusia	Quasi-experimental study	Low	n = 249 patients (171 in the control group and 78 in the intervention group) over 30 years of age	Sociodemographic data (age and sex) and health data (body mass index, presence of hypertension, retinopathy, neuropathy and risk of diabetic foot).	Once the intervention was applied, HbA1c levels were reduced in both groups. The favourable group in this study was the intervention group, since they managed to reduce the levels of glycosylated haemoglobin.
Vilafranca Cartagena et al., 2022 [62]	DM2	Catalonia	Qualitative study	Low	n = 10 adult patients between 55 and 79 years old who had been diagnosed with DM2	The inclusion criteria were adults with DM2 in the age range of 55 to 79 years who had been diagnosed at least two years earlier, presenting complications associated with DM2, good metabolic control and good adherence to healthy treatments.	The study demonstrates the importance of the knowledge and empowerment that patients must have when treating the disease.
Represas-Carrera et al., 2021 [63]	DM2	7 Spanish autonomous communities (CEA)	Randomised clinical trial	Low	n = 694 with DM. CG n = 356 GI n = 338 Patients between 45 and 75 years old unhealthy lifestyle habits	The main variable was HbA1c and the secondary variables: Mediterranean diet, diet quality, physical activity, sedentary lifestyle, smoking and quality of life.	The results showed a significant improvement in adherence to the Mediterranean diet, with the intervention group being the ones who followed the diet best. Non-significance was demonstrated for glycaemic control, physical activity, sedentary lifestyle, smoking and quality of life during the study period.
López-Cobo et al., 2022 [64]	DM2	Barcelona	observational study	Low	n = 20,457 patients, registered between 2012 and 2016 Population with DM2 from 30 to 80 years old.	Age, sex, HbA1c, BMI, systolic and diastolic Blood Pressure (BP), smoking, albumin/creatinine ratio, estimated glomerular filtration rate (eGFR) and blood lipids.	Glycemic control was maintained among the studied population. The chronic complications of type 2 DM with significance in the study were: diabetic retinopathy, diabetic neuropathy, heart failure and peripheral vascular disease.

CRC: colorectal cancer. IC: ischaemic cardiopathy. DM2: type 2 diabetes mellitus. CG: control group. GI: intervention group. HTN: hypertension.

## Data Availability

All data generated or analysed during this study are included in this published study. Other information from this study are available from the corresponding author on reasonable request.

## Appendix A

### Formulation of the Question

Our research question was structured using the tools provided by the PICO mnemonic, where “P” corresponds to the participant; “I”, intervention; “C”, comparison or context; and “O”, outcome. In our case, the “P” was the Spanish population with IC, DM2 and CRC problems; “I” was the strategies of prevention and promotion of the diseases; “C” was the statistical analysis of incidence, prevalence and mortality; and “O” was the current evidence-based scenario of IC, DM2 and CRC. Accordingly, we asked the following question: what is the current epidemiological situation in Spain with the most prevalent HNCDs (IC, DM2 and CRC)?

**Table A1:** Search strategy for the high-prevalence non-communicable diseases: colorectal cancer, ischaemic heart disease and type 2 diabetes mellitus.

Diseases	Databases		
	PubMed	ProQuest	Scopus
Colorectal cancer	(colorectal cancer [title/abstract])) OR (colonic neoplasms [Mesh terms])) AND (incidence)) AND (Spain)	(colon cancer AND epidemiology AND (Spain OR Spanish)	colon AND neoplasms AND epidemiology AND Spain
Ischaemic heart disease	(((ischemic heart disease) AND (myocardial ischemia) AND (((incidence) OR (prevalence)) OR (epidemiology) AND (((Spain) OR (Spanish))	(ischemic heart disease AND risk factors AND incidence AND Spain)	ischemic heart disease AND risk factors AND incidence AND Spain
Diabetes mellitus type 2	((((diabetes mellitus type 2) AND (incidence [MeSH Terms]))) AND (epidemiology)) AND (Spain)	(diabetes mellitus type 2) AND (incidence) AND (risks factors) AND (Spain)	diabetes mellitus type 2 AND risks factors AND incidence AND Spain
